# Carotid pulsatile energy fraction surpasses traditional hemodynamic markers in explaining cognitive impairment among hemodialysis patients

**DOI:** 10.1038/s41440-025-02438-y

**Published:** 2025-11-12

**Authors:** Chih-Cheng Wu, Chieh-kai Chan, Chihchen Liao, Ru-Yin Shu, Mu-Yang Hsieh, Jiun Wang, Shao-Yuan Chuang, Hao-Min Cheng

**Affiliations:** 1 Cardiovascular Center, National Taiwan University Hospital, Hsin-Chu Hospital, Hsin-Chu, Taiwan; 2 College of Medicine, National Taiwan University, Taipei, Taiwan; 3 Institute of Biomedical Engineering, National Tsing-Hua University, Hsin-Chu, Taiwan; 4 Institute of Cellular and System Medicine, National Health Research Institutes, Zhunan, Taiwan; 5 Hemodialysis Center, National Taiwan University Hospital, Hsin-Chu Hospital, Hsin-Chu, Taiwan; 6 Ph.D. Program of Interdisciplinary Medicine (PIM), National Yang Ming Chiao Tung University College of Medicine, Taipei, Taiwan; 7 School of Medicine, Fu Jen Catholic University, New Taipei City, ROC Taiwan; 8 Institute of Population Health Science, National Health Research Institute, Miaoli, Taiwan; 9 Division of Faculty Development, Taipei Veterans General Hospital, Taipei, Taiwan; 10 School of Medicine, National Yang Ming Chiao Tung University, Taipei, Taiwan

**Keywords:** Carotid arteries, Cognition disorders, Hemodialysis, Hemodynamics, Pulsatile flow

## Abstract

Cognitive impairment is a frequent yet poorly understood complication of maintenance hemodialysis. We studied 162 thrice-weekly hemodialysis patients and 1858 community adults characterized previously with identical pressure-flow protocols. Group comparisons were conducted following propensity score matching and utilizing ANCOVA, adjusted for age, sex, educational attainment, body mass index, diabetes, and hypertension. On a mid-week non-dialysis day, synchronous applanation tonometry and Doppler ultrasound recorded ascending-aortic and common-carotid waveforms; mean hydraulic energy, pulsatile energy and the pulsatile energy fraction (PEF) were computed for each heartbeat. Global cognition was assessed with the Montreal Cognitive Assessment (MoCA), with scores < 26 indicating impairment. Cognitive dysfunction was present in 39% of 152 hemodialysis patients versus 33% of community adults (Standardized Mean Difference = 0.134). Compared with the Community group, hemodialysis group exhibited a 45% increase in aortic PEF (0.16 ± 0.07 vs 0.11 ± 0.03), and a 74% increase in carotid PEF (0.080 ± 0.04 vs 0.046 ± 0.02). In multivariable models adjusted for age, sex, education level, and body-mass index, carotid PEF displayed the strongest inverse association with MoCA (standardized β = –0.287, *p* < 0.001). Introducing carotid PEF increased the model’s explained variance and rendered pulse pressure and carotid–femoral pulse-wave velocity non-significant. Conversely, higher steady mean carotid energy correlated positively with MoCA (β = +0.158, *p* = 0.030). These findings indicate that cognitive performance in hemodialysis patients is governed less by conventional pressure or stiffness metrics than by the pulsatile energy transmitted to the carotids, positioning carotid PEF as a mechanistic marker and promising therapeutic target for preserving cognition in this vulnerable population.

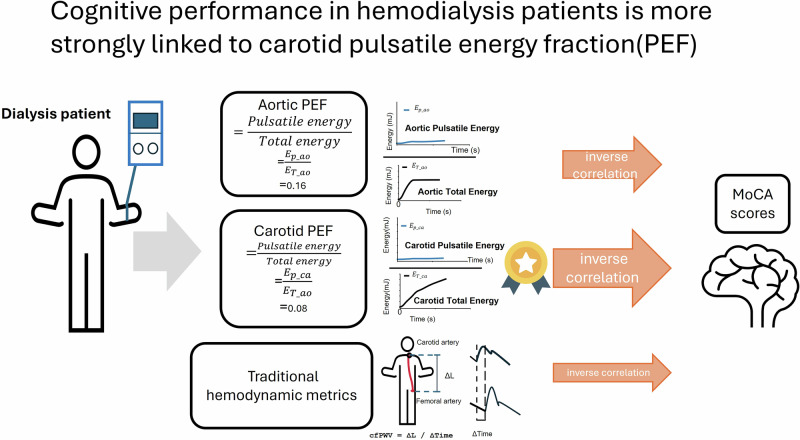

## Introduction

Cognitive dysfunction is a common yet frequently overlooked complication in maintenance-hemodialysis care [1, 2]. Meta-analytic data show that about one patient in every two on chronic dialysis meets criteria for mild cognitive impairment or dementia, a prevalence more than twice that seen in age-matched community [3]. Such deficits are not benign: they are linked to impaired treatment adherence, greater symptom burden and a marked rise in depression and all-cause mortality, with seven-year survival falling from >80% in cognitively intact patients to <50% in those with impairment [4, 5]. Taken together, these observations highlight cognition as a critical, patient-centered outcome of dialysis therapy.

Vascular injury is a primary candidate mechanism; however, traditional hemodynamic surrogates frequently underperform in this population [6]. Hemodialysis patients accumulate advanced arteriosclerosis and medial calcification that stiffen conduit arteries far beyond what is seen in the general ageing process; they also experience large, rapid shifts in intravascular volume and blood pressure lability during and between dialysis sessions [7]. These features blunt the explanatory power of static or single-site markers such as brachial blood pressure, pulse pressure, or carotid–femoral pulse-wave velocity (cf-PWV): heavy calcification distorts PWV by increasing wave speed without proportionally altering distal energy transmission, while sodic hypertensive peaks may leave average clinic BP unchanged [8–10]. In short, traditional metrics record the magnitude of pressure or flow but miss the moment-to-moment hydraulic work that actually impinges on fragile cerebral microvessels.

Recently, an energy-based framework has been proposed to bridge this gap. By integrating simultaneous pressure and flow waveforms, energetic variables such as mean hydraulic energy and the pulsatile energy fraction (PEF) quantify how much steady and pulsatile energy traverses the aorto-carotid interface each heartbeat. In community adults, Cheng et al. showed that carotid PEF and mean energy outperform pulse pressure, flow pulsatility and PWV in predicting Montreal Cognitive Assessment (MoCA) scores, suggesting that energetic load—rather than pressure alone—may be the proximate vascular insult to cognition [11]. However, it remains uncertain whether this concept applies to the particularly vulnerable hemodialysis patients.

Accordingly, our study aimed to compare energy-based hemodynamic indices with traditional surrogates in relation to global cognition in a group of maintenance-hemodialysis patients. We hypothesized that a higher carotid energetic load would show a stronger, independent association with lower MoCA scores than brachial pulse pressure, aortic pressure/flow pulsatility, or PWV.

## Method

### Study participants

The Hsinchu Vascular Study is an ongoing, prospective, multicenter study of maintenance-hemodialysis patients in the Hsinchu District of Taiwan. Adults aged ≥18 years who had received thrice-weekly hemodialysis for at least one month were screened consecutively from 1 January 2022 onward. Patients who had been hospitalized within the preceding month or were excluded. Participation in the hemodynamic substudy was voluntary, as it required an additional outpatient visit of ~1–2 h; about half of the eligible patients consented. Among those who participated, patients with atrial fibrillation or other irregular rhythms, inadequate tonometry waveforms due to technical limitations, or inability to complete cognitive testing because of severe hearing, vision, or speech impairment, or illiteracy beyond the MoCA accommodation were excluded. Of 162 patients with valid hemodynamic data, 152 successfully completed cognitive testing and were included in the cognitive-hemodynamic analysis. Written informed consent was obtained at enrolment, and the protocol was approved by the National Taiwan University Hospital Research Ethics Committee. Baseline demographics, cardiovascular history, medication use, and dialysis vintage were entered into an electronic case-report form (REDCap) and updated every six months. For comparisons, we incorporated a non-dialysis community group drawn from our earlier study on energetic hemodynamics [11]. That investigation enrolled 1858 community-dwelling adults from the Cardiovascular and Disease Risk Factors Two Township Study (CVDFACTS) and the Longitudinal Aging Study of Taipei (LAST) between 2016 and 2020, using identical cognitive testing and central aortic–carotid pressure/flow acquisition protocols as in the present hemodialysis group (Supplementary Table 1). Medication use was not collected in the community group [12].

### Cognitive function

The cognitive function test was performed on a mid-week non-dialysis day in a private room or in as quite an environment as possible. The global cognitive function was evaluated using the MoCA protocol with the Chinese version specifically used in Taiwan, through face-to-face interview by dedicated and qualified nurses’ adherent to the standardized study guide [13]. The MoCA was constituted by 20 items clustered into 7 subgroups, each dedicated to one aspect of cognitive function, namely executive function/visuospatial ability (5 points), attention (6 points), animal naming (3 points), language (3 points), abstraction (2 points), short-term memory (5 points), and orientation (6 points) with a total score of 30 points [14]. Cognitive dysfunction was defined a-priori as MoCA < 26 [15].

### Cardiovascular imaging and waveform acquisition

All ultrasound and tonometry studies were performed on a mid-week, non-dialysis day after participants had rested supine for ≥10 min:

#### Echocardiography

Participants all received transthoracic echocardiography performed by an experienced sonographer. All images were acquired using a commercially available machine (PEFC CVx Ultrasound system, Koninklijke Philips N.V.) and digitized using the TomTec Image-Arena™ Software 4.0 (TomTec Imaging Systems GmbH, Munich, Germany) by the same sonographer. Left-ventricular outflow-tract (LVOT) diameter (parasternal long-axis) and pulsed-wave Doppler velocity (apical five-chamber) were obtained with an PEFC CVx scanner (Philips). Stroke volume, cardiac output and cardiac index were calculated from the LVOT velocity-time integral using standard formulae. The echocardiographic examinations in our study were performed by a board-certified sonographer with 27 years of professional experience in cardiovascular imaging [11].

#### Pressure and flow waveforms

Arterial pressure waveforms were recorded with a high-fidelity applanation tonometer (SPC-350, Millar) [16]. In non-dialysis participants, sequential recordings were obtained from the right common carotid and right femoral arteries. Carotid and aortic flow velocities were sampled with pulsed Doppler (linear-array 3–10 MHz probe positioned 1 cm proximal to the carotid bulb). Diameters were measured at the time of flow sampling and used to convert velocities to volumetric flow.

In hemodialysis patients, tonometry was performed on the carotid, radial, and femoral arteries contralateral to the vascular access side to avoid shunt-related artifacts. Measurements were therefore usually obtained on the right side, unless the patient’s access was on the right arm, in which case the left-sided arteries were examined. Patients with atrial fibrillation or other irregular rhythms were excluded because beat-to-beat variability precludes reliable waveform acquisition. Additional exclusions included inadequate signal quality due to severe vascular calcification, obesity, or technical limitations, and inability to cooperate with positioning because of physical or cognitive impairment, fatigue, or discomfort. Despite these precautions, a small number of patients yielded unusable recordings, and these cases were excluded from the final analysis (see Supplementary Fig. 1).

#### Arterial stiffness

cf-PWV was calculated as surface distance divided by foot-to-foot transit time referenced to the simultaneously recorded ECG. Transit time was calibrated by the simultaneously recorded ECG, and aligned via custom-designed software on a commercial software package (Matlab, version 4.2, The MathWorks, Inc.) [16].

### Hemodynamic analysis

**Fig. 1:**
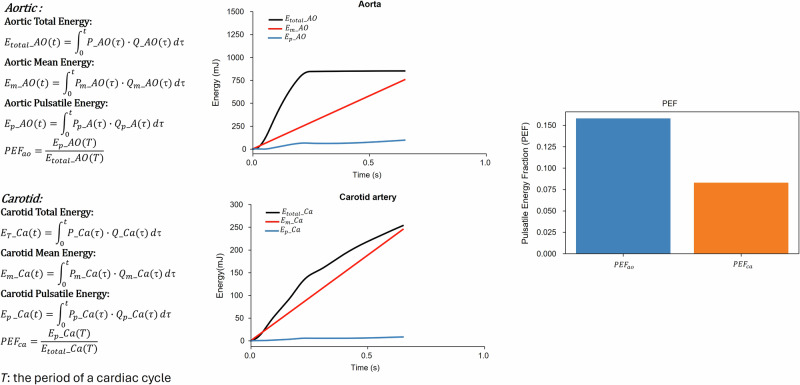
Hemodynamic energy components in the aorta and carotid artery. Panels show the calculation of total, mean, and pulsatile energy in the aorta (top) and carotid artery (bottom). Total energy (E_total_) was obtained by integrating instantaneous pressure–flow products across the cardiac cycle. Mean energy (E_m_) was derived from mean pressure and mean flow, while pulsatile energy (E_p_) reflected the product of pulsatile pressure and pulsatile flow. The pulsatile energy fraction (PEF) was defined as the ratio of pulsatile to total energy at one cardiac cycle (PEF = E_p_/E_total_). Line plots illustrate cumulative energy over time in the aorta (top right) and carotid artery (bottom right), with black, red, and blue lines representing E_total,_ E_m_, and E_p_, respectively. The bar graph compares PEF between the aorta (PEF_ao_) and carotid artery (PEF_ca_), demonstrating higher pulsatile energy transmission in the aorta than in the carotid circulation

Based on our experience in this field, previous studies have confirmed the validity of pressure measurements, showing that noninvasive central BP estimation is reliable for cardiovascular risk assessment [17, 18]. Extending to flow, our studies have shown that carotid velocity and pulsatility are associated with cerebrovascular outcomes and stroke risk [19, 20]. Carotid pressure was calibrated with brachial mean and diastolic values and used as a surrogate for ascending-aortic pressure. Synchronous pressure and flow waveforms were ensemble-averaged over ten beats. We calculated the hydraulic mean, pulsatile energy, and total energy for one cardiac cycle, consistent with those power-based parameters adopted by Haidar et al. [21]. We first separated the measured pressure P (t) and blood flow Q (t) waveforms into their respective mean and pulsatile components: *P(t) = P* + *P*_*p*_ *(t)*, *Q(t)=Q* + *Q*_*p*_ *(t)*. Mean energy, pulsatile energy, total energy were derived for each cardiac cycle by integrating the product *P(t) × Q(t)*, exactly as described by Cheng et al. [11] (Fig. 1). Since the cardiac output is known to be a function of body-size, the total energy, pulsatile energy and mean energy are most likely all associated with patients’ body-size. Therefore, it is sensible to adopt the ratio of the pulsatile energy to total energy ${{E}}_{p}$/${E}_{T}$, e.g. pulsatile energy fraction as a hemodynamic index for assessing terminal organ injury. The same algorithm was applied to aortic and carotid data. Characteristic impedances of the proximal aorta (Z_Ao_) were obtained in the frequency domain following that report. All indices in the present study were computed in a standardized batch-processing manner to minimize operator variability.

### Statistical analysis

We conducted 1:4 propensity score matching, yielding 130 participants in the hemodialysis population and 456 participants in the community population (Table 1). Following matching, analysis of covariance (ANCOVA) was employed to assess group differences, adjusting for age, sex, body mass index, years of education, diabetes mellitus, and hypertension. Continuous variables are presented as mean ± SD or median (IQR); categorical variables as number (percentage). The associations between MoCA score and hemodynamic indices was initially assessed through univariate linear regression to identify the covariates for the multivariable model, including age, sex, body mass index, and educational attainment. As sensitivity analyses, we further repeated the regression after excluding patients with a history of stroke, and added shunt side (left vs right arm access) as a covariate in the full models. These checks were performed to assess the robustness of the associations between hemodynamic parameters and MoCA. To evaluate whether the association of carotid PEF with MoCA was independent of other hemodynamic measures, we performed pairwise multivariable linear regression models, entering carotid PEF together with a single comparator parameter (e.g., cf-PWV, brachial pulse pressure, carotid mean energy). Each model was adjusted for age, sex, education level, and BMI. Two-tailed *p* < 0.05 denoted statistical significance unless stated otherwise. Analyses were performed with SPSS v24.0.

**Table 1:** Baseline characteristics of the matched group

Variables	Hemodialysis	Community	SMD
Mean (SD), *n* (%), or median (IQR)	Group	Group	
*N*	130	456	
Age (years)^a^	64.5 (11.7)	64.3 (10.5)	0.020
Male, *n* (%)^a^	78 (60)	243 (53)	0.138
Education level, *n* (%)^a^			0.172
Elementary school/below	20 (15)	71 (16)	
Junior school	32 (25)	82 (18)	
High school	50 (38)	129 (28)	
University or higher	28 (22)	174 (38)	
Clinical characteristics			
BMI, kg/m^2^	23.9 (3.5)	24.1 (3.2)	0.054
Hemodialysis duration, y	4.6 (1.2, 4.6)		
Medical history, *n* (%)			
Hypertension	110 (85)	186 (41)	1.064
Diabetes mellitus	72 (55)	60 (13)	1.024
Biochemistry data			
Cholesterol, mg/dl	149.1 (37.4)	199.7 (36.5)	1.357
Albumin, g/dl	3.9 (0.25)	NA	
Calcium x Phosphate	49.4 (14.3)	NA	
Kt/V	1.61 (0.24)	NA	
Medications, *n* (%)			
Antiplatelet	23 (18)	NA	
Statin	48 (37)	NA	
RAI	47 (36)	NA	
CCB	49 (38)	NA	
Brachial BP			
Systolic BP, mmHg	133.7 (26.6)	129.3 (17.7)	0.197
Diastolic BP, mmHg	74.9 (15.1)	76.2 (9.8)	0.105
Pulse pressure, mmHg	58.9 (17.2)	53.1 (13.3)	0.389
Heart rate, /min	72.1 (21.9)	68.6 (12.6)	0.201
Blood vessel function			
cfPWV, m/sec	13.5 (5.9)	13.5 (3.9)	0.002
Zao, dyne*s/cm^5^	261.1 (178.7)	128.7 (61.9)	0.99
Zca, dyne*s/cm^5^	1325.5 (605.4)	1211 (523.2)	0.196
Cognitive function			0.134
Cognitive dysfunction, *n* (%)	51 (39)	151 (33)	
MoCA score, median (IQR)	26 (24, 29)	27 (25, 28)	0.075

Values are expressed by *n* (%), mean (SD), or median (IQR) as appropriate

Data are from the hemodialysis participants who completed hemodynamic assessment (*n* = 162)

Of these, 152 completed cognitive testing and are analyzed in Tables 3 and 4

*BP* blood pressure, *cf-PWV* carotid-femoral pulse wave velocity, *Kt/V* urea clearance, *Zao* Aortic characteristic impedance, *Zca* Carotid characteristic impedance, *MoCA* Montreal Cognitive Assessment; Cognitive dysfunction: MoCA score < 26, *SMD* standardized mean difference

^a^Variables included in the propensity score (PS) model

## Results

### Baseline characteristics

The baseline characteristics of the original study populations and the propensity score matched groups are detailed in Supplementary Table 1 and Table 1, respectively. A total of 130 patients receiving long-term hemodialysis were assessed following matching (78 men and 52 women; Table 1). The mean age was 64.5 ± 11.7 years and the median dialysis vintage 4.6 years (inter-quartile range 1.2–4.6). Compared with community participants, the Hemodialysis patients and controls were similar in age (64.5 ± 11.7 vs 64.3 ± 10.5, SMD = 0.02), while the proportion of males was 60% in the hemodialysis group and 50% among controls. Cardiometabolic burden was heavier in hemodialysis group (hypertension 85% vs 41%; diabetes 55% vs 13%; SMD = 1.064 and 1.024). Five of the hemodialysis group have history of previous stroke. Cognitive impairment (MoCA <26) was slightly higher in hemodialysis patients compared with controls (39% vs 33%; SMD = 0.134). with a one-point lower median MoCA score (26 vs 27). Among the 162 patients with valid hemodynamic recordings, 152 completed MoCA and were included in the cognitive–hemodynamic analyses.

### Hemodynamic parameters

**Fig. 2:**
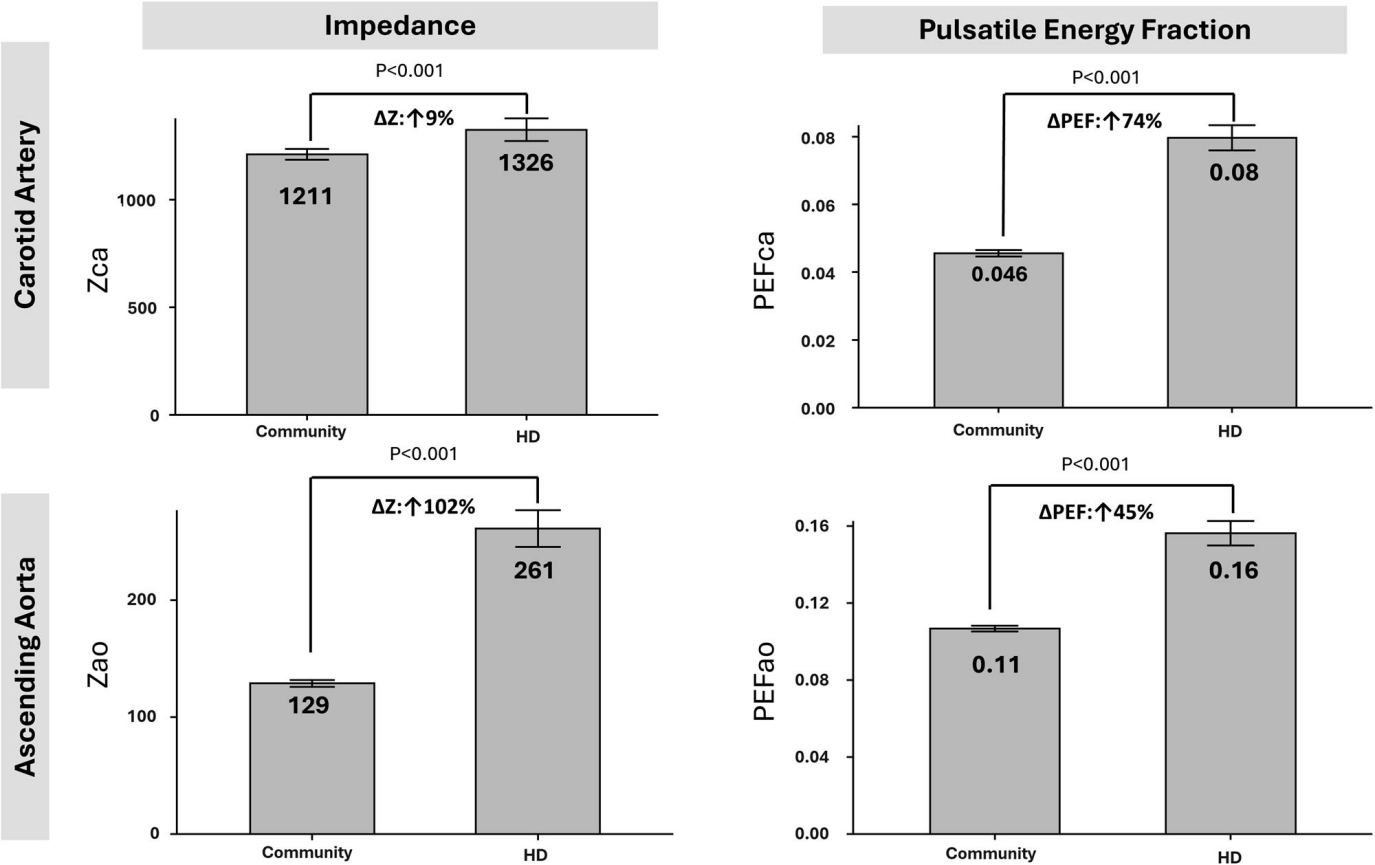
Characteristic impedance and pulsatile energy fraction in hemodialysis (HD) patients versus community controls. The left panels depict mean characteristic impedance (Z) at the common carotid artery (Z_ca_, upper-left) and ascending aorta (Z_ao_, lower-left). The right panels show mean pulsatile energy fraction (PEF = pulsatile/total hydraulic energy) at the same vascular sites (PEF_ca_ and PEF_ao_). Black bars represent the means of impedance and PEF of HD patients and community controls. Percentage differences (Δ) denote the relative increase in HD patients compared with community controls: carotid Z + 9%, aortic Z + 102%, carotid PEF + 74%, and aortic PEF + 45%. Values are expressed in dyne·s·cm⁻⁵ for impedance and are dimensionless for PEF. The *p*-value was calculated using ANCOVA, controlling for age, sex, body mass index, years of education, diabetes mellitus, and hypertension

Hemodynamic characteristics are summarized in Table 2. All tonometry recordings were obtained contralateral to the vascular access side. Brachial BP used in covariates was also taken on the non-access arm per standard practice. Mean aortic flow did not differ between groups, but pulsatile indices were consistently amplified in hemodialysis group: peak flow was 8.6% higher (350 ± 101 vs 322 ± 74 ml s^−1^, *p* < 0.001), and total, mean and pulsatile hydraulic energies were all significantly greater, culminating in a 45% higher pulsatile energy fraction (0.16 ± 0.07 vs 0.11 ± 0.03; all *p* < 0.001). Although mean carotid flow was comparable (22.9 ± 6.6 vs 23.4 ± 5.4 ml s⁻¹; *p* = 0.164), hemodialysis patients exhibited markedly greater pulsatile load: peak flow and flow pulsatility index were higher (1.81 ± 0.43 vs 1.37 ± 0.31; *p* < 0.001), and pulsatile carotid energies were elevated, with increased carotid PEF (0.080 ± 0.04 vs 0.046 ± 0.02; *p* < 0.001; Fig. 2. Although total and mean energy did not differ between groups, pulsatile energy was significantly higher in the hemodialysis cohort.

**Table 2:** Flow and energy related parameters at ascending aorta and common carotid arteries

Variables mean (SD)	Hemodialysis group	Community group	*P* value
*N*	130	456	
Ascending aorta			
Mean flow, ml/s	84.0 (24.4)	82.3 (22.0)	0.538
Peak flow, ml/s	350.0 (101.0)	321.8 (77.4)	<0.001
Flow pulsatility index	4.21 (0.51)	3.98 (0.61)	<0.001
Total energy, mJ	1212.3 (659.6)	1041.4 (303.0)	0.008
Mean energy, mJ	1016.4 (562.1)	927.5 (261.4)	0.195
Pulsatile energy, mJ	195.9 (144.3)	113.8 (57.2)	<0.001
Pulsatile energy fraction	0.16 (0.07)	0.11 (0.03)	<0.001
Common carotid artery			
Mean flow, ml/s	22.9 (6.6)	23.4 (5.4)	0.164
Peak flow, ml/s	41.1 (14.7)	32.1 (10.2)	<0.001
Flow pulsatility index	1.81 (0.43)	1.37 (0.31)	<0.001
Total energy, mJ	307.5 (185.8)	279.6 (80.2)	0.405
Mean energy, mJ	281.9 (169.9)	266.6 (75.5)	0.919
Pulsatile energy, mJ	25.6 (20.8)	13.0 (7.6)	<0.001
Pulsatile energy fraction	0.080 (0.04)	0.046 (0.02)	<0.001

*P* values between groups were estimated using ANCOVA models, adjusting for age, education level, sex, body mass index, diabetes, and hypertension

### Clinical and hemodynamic correlates of cognitive function

Exploratory univariable associations of MoCA with baseline clinical variables are provided in Table 2. In the multivariable analyses adjusted for age, sex, BMI, and education level, several energetic parameters remained significantly related to MoCA (Table 3). The carotid PEF demonstrated the strongest independent association with lower MoCA scores (β = –0.287, *p* < 0.001), whereas carotid mean energy showed a weaker but positive association (β = 0.158, *p* = 0.030). In contrast, traditional surrogates such as brachial pulse pressure, aortic flow pulsatility, and cf-PWV were attenuated and no longer significant after adjustment. Overall, carotid artery–based hemodynamic measures, particularly the carotid PEF, exhibited more robust and consistent associations with cognitive performance than pressure- or flow-based indices in either the carotid or aorta.

**Table 3:** Associations among hemodynamic parameters with MoCA score

**Variables**	**Crude**		**Adjusted**	
	β	*P* value	β	*P* value
cf-PWV	−0.232	0.006	−0.143	0.095
Brachial artery				
Systolic pressure, mmHg	−0.045	0.582	−0.135	0.078
Diastolic pressure, mmHg	0.166	0.041	−0.043	0.612
Mean pressure, mmHg	0.086	0.292	−0.094	0.326
Pulse pressure, mmHg	−0.376	<0.001	−0.280	0.001
Ascending aorta				
Mean flow, ml/s	0.118	0.147	0.034	0.651
Peak flow, ml/s	0.032	0.695	−0.072	0.339
Flow pulsatility index	−0.066	0.420	0.009	0.904
Total energy, MJ	0.092	0.260	0.092	0.206
Mean energy, MJ	0.151	0.064	0.121	0.097
Pulsatile energy, mJ	−0.167	0.040	−0.122	0.139
Pulsatile energy fraction	−0.331	<0.001	−0.178	0.028
Common carotid arteries				
Mean flow, ml/s	0.220	0.007	0.113	0.135
Peak flow, ml/s	−0.134	0.100	−0.142	0.055
Flow pulsatility index	−0.218	0.007	−0.126	0.112
Total energy, MJ	0.163	0.045	0.141	0.052
Mean energy, mJ	0.193	0.017	0.158	0.030
Pulsatile energy, mJ	−0.127	0.118	−0.174	0.035
Pulsatile energy fraction	−0.420	<0.001	−0.287	<0.001

*β* standardized β regression coefficient, *Adjusted* adjusted for age, sex, education level, body mass index

### Multivariable analyses and comparative analyses

In multivariable models adjusted for age, sex, body mass index, and education level, the carotid PEF showed the strongest association with cognitive function (β = –0.287, *p* < 0.001). The next most significant correlate was brachial artery pulse pressure (β = –0.280, *p* = 0.001) (Table 3). Sensitivity analyses are shown in Supplementary Table 3. Excluding the 5 patients with prior stroke (*n* = 147) or adding shunt side as an additional covariate did not materially alter the associations between hemodynamic parameters and MoCA, confirming the robustness of the primary findings. To further compare the relative contributions of hemodynamic parameters, we conducted pairwise multivariable regression analyses, entering carotid PEF together with a single comparator metric in each model (Table 4). Carotid PEF remained independently associated with lower MoCA scores across all pairwise models. Specifically, it outperformed the carotid flow pulsatile index (model 1), aortic PEF (model 2), carotid mean energy (model 3), and brachial pulse pressure (model 4), whereas the comparator parameters were not significant.

**Table 4:** The comparative association analysis among hemodynamic parameters and MoCA scores

Regression analysis for MoCA score	β	95% CI (LL)	(UL)	*P* value
Model 1				
Carotid pulsatile energy fraction	−0.419	−55.016	−25.371	<0.001
Carotid flow pulsatility index	−0.003	−0.013	0.012	0.966
Model 2				
Carotid pulsatile energy fraction	−0.411	−53.279	−25.574	<0.001
Carotid energy mean energy	0.171	0.001	0.008	0.020
Model 3				
Carotid pulsatile energy fraction	−0.538	−79.736	−23.375	<0.001
Aortic pulsatile energy fraction	0.135	−9.495	25.737	0.364
Model 4				
Carotid pulsatile energy fraction	−0.376	−56.924	−22.877	<0.001
cfPWV	−0.124	−0.219	0.028	0.128
Model 5				
Carotid pulsatile energy fraction	−0.308	−49.233	−9.858	0.004
Brachial artery pulse pressure	−0.159	−0.088	0.011	0.128

Each row represents a multivariable linear regression model including carotid pulsatile energy fraction(PEF) and one comparator parameter as independent variables, with MoCA as the dependent variable. All models adjusted for age, sex, education level, and BMI

*β* standardized coefficient, *CI* confidence interval

## Discussion

### Main findings

Our study demonstrates that cognitive dysfunction, prevalent in maintenance hemodialysis patients, is more closely related to the pulsatile energy burden delivered to the cerebral circulation than to conventional hemodynamic surrogates. By directly comparing our hemodialysis group with a large community-based group from our prior studies, we confirmed that dialysis patients experience a disproportionate amplification of carotid energy pulsatility, despite only modest differences in steady flow [11]. Among all assessed parameters, the carotid PEF, a composite measure integrating pressure and flow, showed the strongest and independent association with lower MoCA scores. Traditional markers, including brachial pulse pressure, aortic flow pulsatility, and cf-PWV, lost significance once energetic indices were included. These results highlight that the transmission of pulsatile energy from a stiffened aorta into the cerebral circulation, rather than pressure or stiffness alone, may be a key hemodynamic mechanism of cognitive impairment in hemodialysis patients.

### Characteristic hemodynamic changes in dialysis patients

Our hemodialysis group displayed a distinct “high-impedance, high-energy” vascular phenotype compared with age-matched community controls. C–f PWV was only marginally higher (13.3 ± 5.8 vs 12.2 ± 3.6 m s^−1^, *p* = 0.001), offering limited discriminatory power; however, the characteristic aortic impedance (Zₐₒ) was doubled (256 ± 170 vs 125 ± 62 dyne·s·cm^−5^, *p* < 0.001), indicating a marked flattening of the normal aorto-carotid impedance “step-up” [22]. With this protective gradient eroded, a larger fraction of pulsatile energy is able to penetrate the carotid circulation—reflected by the higher proportion of aortic pulsatile energy reaching the carotid arteries in hemodialysis patients (13% vs 11%).

Energetic indices echoed these structural shifts. Aortic PEF increased by nearly 45% (0.16 ± 0.07 vs 0.11 ± 0.03), while carotid PEF was 74% higher (0.080 ± 0.04 vs 0.046 ± 0.02) despite nearly identical mean carotid flow (22.9 ± 6.6 vs 23.4 ± 5.4 ml s⁻¹). This amplification of pulsatile hydraulic energy is plausibly driven by a constellation of dialysis-specific factors: medial calcification and collagen cross-linking that stiffen the proximal aorta, chronic hyper-circulation that augments stroke volume, and the large, rapid fluid shifts inherent to thrice-weekly ultrafiltration [23]. Collectively, these processes stiffen the aortic root more than downstream carotid arteries, compress the impedance mismatch, and permit injurious pressure–flow oscillations to surge into the cerebral microvasculature. These mechanistic changes help explain both the high burden of cognitive dysfunction and the limited explanatory value of traditional markers such as brachial blood pressure or PWV in the hemodialysis population.

Our data demonstrate a higher carotid pulsatile energy fraction (Ep/ET) in hemodialysis patients, suggesting that pulsatile wave energy is more prone to transmit across the aortic–carotid junction. Supplementary Fig. 2 provides a schematic illustration of this mechanism.

### Possible mechanisms of pulsatile brain injury

High carotid PEF quantifies the share of hydraulic power delivered as rapid pressure-flow oscillations, and our data confirm that this pulsatile excess, rather than the steady component, tracks cognitive loss in dialysis. In fully-adjusted models, a 1-SD rise in carotid PEF (β = –0.287) translated into roughly a 1-point lower MoCA score, whereas one SD increase in mean carotid energy (β = +0.158) corresponded to around 0.5-point MoCA gain, underscoring the protective role of continuous perfusion. Laboratory studies provide a mechanistic bridge: when penetrating arterioles are perfused with unchanged mean flow but tripled pulsatile energy, cyclic wall strain doubles, endothelial tight junctions rupture, and perivascular astrocytes activate [24, 25]. These lesions mirror in-vivo imaging findings that relate high PEF, more strongly than systolic pressure, flow pulsatility, or cf-PWV to deep-white-matter hypoperfusion, lacunes, and blood-brain-barrier leakage [25, 26]. Mathematically, pressure measures distending force and flow measures volume, but their time-integrated product, energy, captures the moment-to-moment mechanical work absorbed by the vessel wall; sharp energy surges impose shear and stretch unavailable to either signal alone. Repeated bursts of such power activate endothelial NADPH-oxidase and NF-κB, up-regulating ICAM-1, VCAM-1, and pro-inflammatory cytokines; in uremia this cascade is amplified, further weakening the blood–brain barrier and permitting neurotoxic proteins and immune cells to invade the parenchyma [27, 28]. Thus, when blood vessels become stiff and lose their natural damping, the pressure pulses that normally maintain blood flow turn harmful, damaging small vessels and impairing cognition, an injury that traditional pressure- or flow-based measures miss but energy-based indices reveal.

### Other relevant hemodynamic markers

Brachial pulse pressure remains a practical bedside surrogate because it is inexpensive, widely available and, before energetic variables are added, retains independent predictive value for cognition. In the fully-adjusted model (Table 3) each SD rise in pulse pressure was associated with a 0.28-SD drop in MoCA (β = –0.280, *p* = 0.001). Mechanistically, both pulse pressure and carotid PEF measure arterial pulsatility, but they interrogate different facets of the pulse: pulse pressure reflects the amplitude of the pressure wave, whereas PEF captures the time-integrated product of pressure and flow, the actual mechanical work absorbed by cerebral arterioles. Accordingly, when carotid PEF and pulse pressure were entered together (Table 4), the explanatory power of pulse pressure fell (β = –0.159, *p* = 0.128), indicating that pulse pressure registers only part of the hemodynamic insult.

Even so, pulse pressure serves as a pragmatic screening marker that can flag dialysis patients who may benefit from deeper hemodynamic profiling. Therapeutically, interventions that blunt the central pressure wave (such as volume optimization, β-blockade, or renin–angiotensin antagonism) are expected to lower both pulse pressure and PEF, whereas strategies that improve impedance matching or modulate flow (such as aortic compliance enhancers and arteriovenous-fistula banding) may preferentially reduce PEF while leaving pulse pressure largely unchanged [29–33]. Thus, pulse pressure can be viewed as an accessible gateway metric, while carotid PEF offers a more mechanistic target for precision interventions aimed at protecting the dialysis brain.

### Strength and limitations

The present analysis has several strengths. First, it is the largest hemodynamic study to date in a dialysis population that combines beat-to-beat pressure and flow at both the aorta and carotid artery, allowing derivation of truly energy-based indices rather than relying on inferred waveforms. Second, all recordings were obtained prospectively on a mid-week, non-dialysis day under standardized conditions, reducing the confounding influence of acute volume shifts. Third, cognitive status was assessed face-to-face with the MoCA by trained nurses, minimizing misclassification and enhancing external validity for everyday clinical screening.

This study has limitations. Its cross-sectional design precludes causal inference; whether lowering carotid energetic load slows cognitive decline requires longitudinal or interventional studies. The sample, although multicenter, was drawn from a single geographic region and may not capture the full ethnic or practice variability of global dialysis populations. MoCA, while practical, provides only a global score and may miss domain-specific deficits; more detailed neuropsychological testing and brain imaging would have provided greater precision. Participation in the tonometry substudy was voluntary and required an additional outpatient visit, so only about half of eligible patients participated, raising the possibility of selection bias. Although baseline features were broadly similar to the overall Hsinchu Vascular Study participants, residual differences cannot be excluded. Patients with prior stroke were not excluded, but sensitivity analyses omitting these cases yielded consistent results. Cancer history was not collected, leaving potential for unmeasured confounding. Finally, all tonometry measurements were obtained contralateral to the vascular access, minimizing but not fully excluding shunt-related effects on central hemodynamics.

### Clinical perspectives

Despite these caveats, the findings carry clear clinical implications. They suggest that routine surveillance of hemodynamic energy, not merely pressure or could identify patients at greatest cognitive risk and open a new therapeutic target: attenuating the pulsatile energy that reaches the brain. Whether through optimized volume management, heart-rate control, pharmacological augmentation of aortic compliance, or dialysis-machine algorithms that blunt intradialytic pressure swings, strategies that lower carotid PEF may offer a tractable route to preserve cognition in this vulnerable population.

## Conclusion

Cognitive impairment affects more than one-third of patients receiving maintenance hemodialysis and is linked far more strongly to the pulsatile energy delivered to the carotid circulation than to traditional surrogates such as brachial blood pressure, cf–PWV, or aortic flow pulsatility. Whereas higher steady carotid energy appears beneficial, a raised carotid pulsatile energy fraction marks injurious oscillatory work that the stiff, volume-fluctuating dialysis vasculature can no longer buffer. Brachial pulse pressure remains a convenient bedside screen, but energetic indices provide a clearer mechanistic target. Strategies that attenuate pulsatile energy, now warrant prospective evaluation to protect cognition in this vulnerable population.

## Supplementary information

Supplementary information

## Data Availability

The article’s data will be shared at reasonable request to the corresponding author after one year after publication.

## References

[1] Sarnak MJ, Tighiouart H, Scott TM, Lou KV, Sorensen EP, Giang LM, et al. Frequency of and risk factors for poor cognitive performance in hemodialysis patients. Neurology. 2013;80:471. 23303848 10.1212/WNL.0b013e31827f0f7f PMC3590049

[2] Murray AM, Tupper DE, Knopman DS, Gilbertson DT, Pederson SL, Li S, et al. Cognitive impairment in hemodialysis patients is common. Neurology. 2006;67:216. 16864811 10.1212/01.wnl.0000225182.15532.40

[3] Zhang J, Wu L, Wang P, Pan Y, Dong X, Jia L, et al. Prevalence of cognitive impairment and its predictors among chronic kidney disease patients: a systematic review and meta-analysis. PLoS One. 2024;19:e0304762. 38829896 10.1371/journal.pone.0304762 PMC11146742

[4] Griva K, Stygall J, Hankins M, Davenport A, Harrison M, Newman SP. Cognitive impairment and 7-year mortality in dialysis patients. Am J Kidney Dis. 2010;56:693–703. 20800327 10.1053/j.ajkd.2010.07.003

[5] Sorensen EP, Sarnak MJ, Tighiouart H, Scott T, Giang LM, Kirkpatrick B, et al. The kidney disease quality of life cognitive function subscale and cognitive performance in maintenance hemodialysis patients. Am J Kidney Dis. 2012;60:417–26. 22425261 10.1053/j.ajkd.2011.12.029 PMC3547669

[6] Miglinas M, Cesniene U, Janusaite MM, Vinikovas A. Cerebrovascular disease and cognition in chronic kidney disease patients. Front Cardiovasc Med. 2020;7:2020. 10.3389/fcvm.2020.00096 PMC7283453 32582768

[7] Meeus F, Kourilsky O, Guerin AP, Gaudry C, Marchais SJ, London GM. Pathophysiology of cardiovascular disease in hemodialysis patients. Kidney Int. 2000;58:S140–S7. 10.1046/j.1523-1755.2000.07618.x 10936811

[8] Aimagambetova B, Ariko T, Merritt S, Rundek T. Arterial stiffness measured by pulse wave velocity correlated with cognitive decline in hypertensive individuals: a systematic review. BMC Neurol. 2024;24:393. 39415095 10.1186/s12883-024-03905-8 PMC11481605

[9] Kim ED, Meoni LA, Jaar BG, Shafi T, Linda Kao WH, Estrella MM, et al. Association of arterial stiffness and central pressure with cognitive function in incident hemodialysis patients: the PACE study. Kidney Int Rep. 2017;2:1149–59. 29270523 10.1016/j.ekir.2017.07.013 PMC5733684

[10] Giang LM, Tighiouart H, Lou KV, Agganis B, Drew DA, Shaffi K, et al. Measures of blood pressure and cognition in dialysis patients. Hemodial Int Int Symp Home Hemodial. 2013;17:24–31. 10.1111/j.1542-4758.2012.00718.x PMC4076621 22716218

[11] Cheng HM, Wang JJ, Chuang SY, Lin CH, Mitchell GF, Huang CJ, et al. Dissecting the vascular-cognitive nexus: energetic vs. conventional hemodynamic parameters. Hypertens Res. 2024;47:2262–74. 38982290 10.1038/s41440-024-01735-2 PMC11374758

[12] Yeh CJ, Pan WH, Jong YS, Kuo YY, Lo CH. Incidence and predictors of isolated systolic hypertension and isolated diastolic hypertension in Taiwan. J Formos Med Assoc. 2001;100:668–75. 11760372

[13] Tsai JC, Chen CW, Chu H, Yang HL, Chung MH, Liao YM, et al. Comparing the sensitivity, specificity, and predictive values of the montreal cognitive assessment and mini-mental state examination when screening people for mild cognitive impairment and dementia in Chinese population. Arch Psychiatr Nurs. 2016;30:486–91. 27455923 10.1016/j.apnu.2016.01.015

[14] Folstein MF, Folstein SE, McHugh PR. “Mini-mental state”. A practical method for grading the cognitive state of patients for the clinician. J Psychiatr Res. 1975;12:189–98. 1202204 10.1016/0022-3956(75)90026-6

[15] Nasreddine ZS, Phillips NA, Bedirian V, Charbonneau S, Whitehead V, Collin I, et al. The Montreal Cognitive Assessment, MoCA: a brief screening tool for mild cognitive impairment. J Am Geriatr Soc. 2005;53:695–9. 15817019 10.1111/j.1532-5415.2005.53221.x

[16] Lin YP, Chen CH, Hsu TL, Yang WC, Ding PY. Sequential tonometry as a practical method to estimate truncal pulse wave velocity. Zhonghua Yi Xue Za Zhi. 2001;64:693–702. 11922488

[17] Chen CH, Nevo E, Fetics B, Pak PH, Yin FC, Maughan WL, et al. Estimation of central aortic pressure waveform by mathematical transformation of radial tonometry pressure. Validation of generalized transfer function. Circulation. 1997;95:1827–36. 9107170 10.1161/01.cir.95.7.1827

[18] Cheng HM, Chuang SY, Sung SH, Yu WC, Pearson A, Lakatta EG, et al. Derivation and validation of diagnostic thresholds for central blood pressure measurements based on long-term cardiovascular risks. J Am Coll Cardiol. 2013;62:1780–7. 23850921 10.1016/j.jacc.2013.06.029 PMC3884552

[19] Chuang SY, Cheng HM, Bai CH, Yeh WT, Chen JR, Pan WH. Blood pressure, carotid flow pulsatility, and the risk of stroke: a community-based study. Stroke. 2016;47:2262–8. 27491737 10.1161/STROKEAHA.116.013207

[20] Chuang SY, Wang PN, Chen LK, Chou KH, Chung CP, Chen CH, et al. Associations of blood pressure and carotid flow velocity with brain volume and cerebral small vessel disease in a community-based population. Transl Stroke Res. 2021;12:248–58. 32737795 10.1007/s12975-020-00836-7

[21] Haidar MA, van Buchem MA, Sigurdsson S, Gotal JD, Gudnason V, Launer LJ, et al. Wave reflection at the origin of a first-generation branch artery and target organ protection: the AGES-Reykjavik study. Hypertension. 2021;77:1169–77. 33689461 10.1161/HYPERTENSIONAHA.120.16696 PMC9395164

[22] Vatner SF, Zhang J, Vyzas C, Mishra K, Graham RM, Vatner DE. Vascular stiffness in aging and disease. Front Physiol. 2021;12:762437. 34950048 10.3389/fphys.2021.762437 PMC8688960

[23] Wanner C, Amann K, Shoji T. The heart and vascular system in dialysis. Lancet. 2016;388:276–84. 27226133 10.1016/S0140-6736(16)30508-6

[24] Baumbach GL. Effects of increased pulse pressure on cerebral arterioles. Hypertension. 1996;27:159–67. 8567036 10.1161/01.hyp.27.2.159

[25] Garcia-Polite F, Martorell J, Del Rey-Puech P, Melgar-Lesmes P, O’Brien CC, Roquer J, et al. Pulsatility and high shear stress deteriorate barrier phenotype in brain microvascular endothelium. J Cereb Blood Flow Metab. 2017;37:2614–25. 27702879 10.1177/0271678X16672482 PMC5531355

[26] Conley AC, Karayanidis F, Jolly TAD, Yang MH, Hsieh S. Cerebral arterial pulsatility and global white matter microstructure impact spatial working memory in older adults with and without cardiovascular risk factors. Front Aging Neurosci. 2020;12:245. 32848715 10.3389/fnagi.2020.00245 PMC7427001

[27] Cheng JJ, Wung BS, Chao YJ, Wang DL. Cyclic strain-induced reactive oxygen species involved in ICAM-1 gene induction in endothelial cells. Hypertension. 1998;31:125–30. 9449403 10.1161/01.hyp.31.1.125

[28] Baaten C, Vondenhoff S, Noels H. Endothelial cell dysfunction and increased cardiovascular risk in patients with chronic kidney disease. Circ Res. 2023;132:970–92. 37053275 10.1161/CIRCRESAHA.123.321752 PMC10097498

[29] Loutradis C, Papagianni A, Ekart R, Theodorakopoulou M, Minopoulou I, Pagourelias E, et al. Excess volume removal following lung ultrasound evaluation decreases central blood pressure and pulse wave velocity in hemodialysis patients: a LUST sub-study. J Nephrol. 2020;33:1289–300. 32447618 10.1007/s40620-020-00745-w

[30] Lim S, Kook H, Yu CW, Kim JH, Cha JJ, Joo HJ, et al. Differential effects of antihypertensive drugs on central blood pressure: a randomized comparison between nebivolol and telmisartan. Am J Hypertens. 2025;20250610, 10.1093/ajh/hpaf098. 10.1093/ajh/hpaf098 40490940

[31] Lacolley P, Safar ME, Regnault V, Frohlich ED. Angiotensin II, mechanotransduction, and pulsatile arterial hemodynamics in hypertension. Am J Physiol Heart Circ Physiol. 2009;297:H1567–75. 19734358 10.1152/ajpheart.00622.2009

[32] Liu AH, Bondonno CP, Croft KD, Puddey IB, Woodman RJ, Rich L, et al. Effects of a nitrate-rich meal on arterial stiffness and blood pressure in healthy volunteers. Nitric Oxide. 2013;35:123–30. 24120618 10.1016/j.niox.2013.10.001

[33] Vaes RH, Tordoir JH, Scheltinga MR. Systemic effects of a high-flow arteriovenous fistula for hemodialysis. J Vasc Access. 2014;15:163–8. 24170583 10.5301/jva.5000196

