# Supplementary material for: Carotid pulsatile energy fraction surpasses traditional hemodynamic markers in explaining cognitive impairment among hemodialysis patients
Source: Hypertens Res. 2025 Nov 12;49(2):396–406. doi: 10.1038/s41440-025-02438-y (PMC12823414; doi:10.1038/s41440-025-02438-y)
Supplement: Supplementary file 1 — Supplementary information [file 41440_2025_2438_MOESM1_ESM.docx]

**Carotid Pulsatile Energy Fraction Surpasses Traditional Hemodynamic Markers in Explaining Cognitive Impairment among Hemodialysis Patients**

Chih-Cheng Wu, MD (1-4); Chieh-kai Chan, MD (2, 5); Chihchen Liao, PhD (6), Ru-Yin Shu, MD (1, 2); Mu-Yang Hsieh, MD, PhD (1,2); Jiun-Jr Wang, PhD (8); Shao-Yuan Chuang, PhD (7); Hao-Min Cheng, MD, PhD (9, 10)

1. Cardiovascular center, National Taiwan University Hospital, Hsin-Chu Hospital, Hsin-Chu, Taiwan
2. College of Medicine, National Taiwan University, Taipei, Taiwan.
3. Institute of Biomedical Engineering, National Tsing-Hua University, Hsin-Chu, Taiwan.
4. Institute of Cellular and System Medicine, National Health Research Institutes, Zhunan, Taiwan
5. Hemodialysis center, National Taiwan University Hospital, Hsin-Chu Hospital, Hsin-Chu, Taiwan
6. Ph.D. Program of Interdisciplinary Medicine (PIM), National Yang Ming Chiao Tung University College of Medicine, Taipei, Taiwan
7. Institute of Population Health Science, National Health Research Institute, Miaoli, Taiwan
8. School of Medicine, Fu Jen Catholic University, New Taipei City, Taiwan, ROC
9. Division of Faculty Development, Taipei Veterans General Hospital, Taipei, Taiwan
10. School of Medicine, National Yang Ming Chiao Tung University, Taipei, Taiwan

**Supplementary Figure 1. *Patient selection flow diagram for the Hsinchu Vascular Study hemodialysis cohort.***


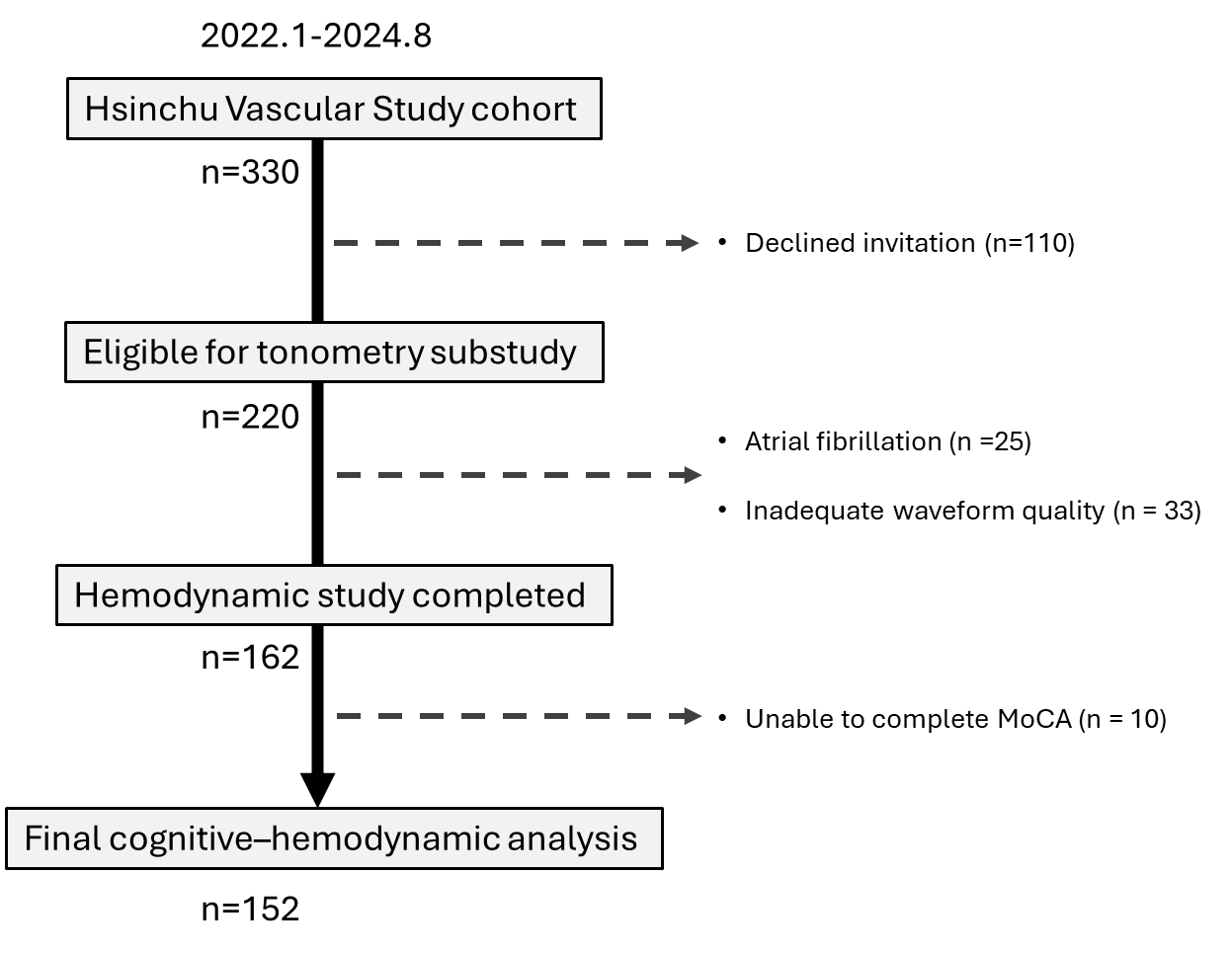


**Supplementary Figure 2 Schematic illustration of the concept of pulsatile energy fraction (PEF).**


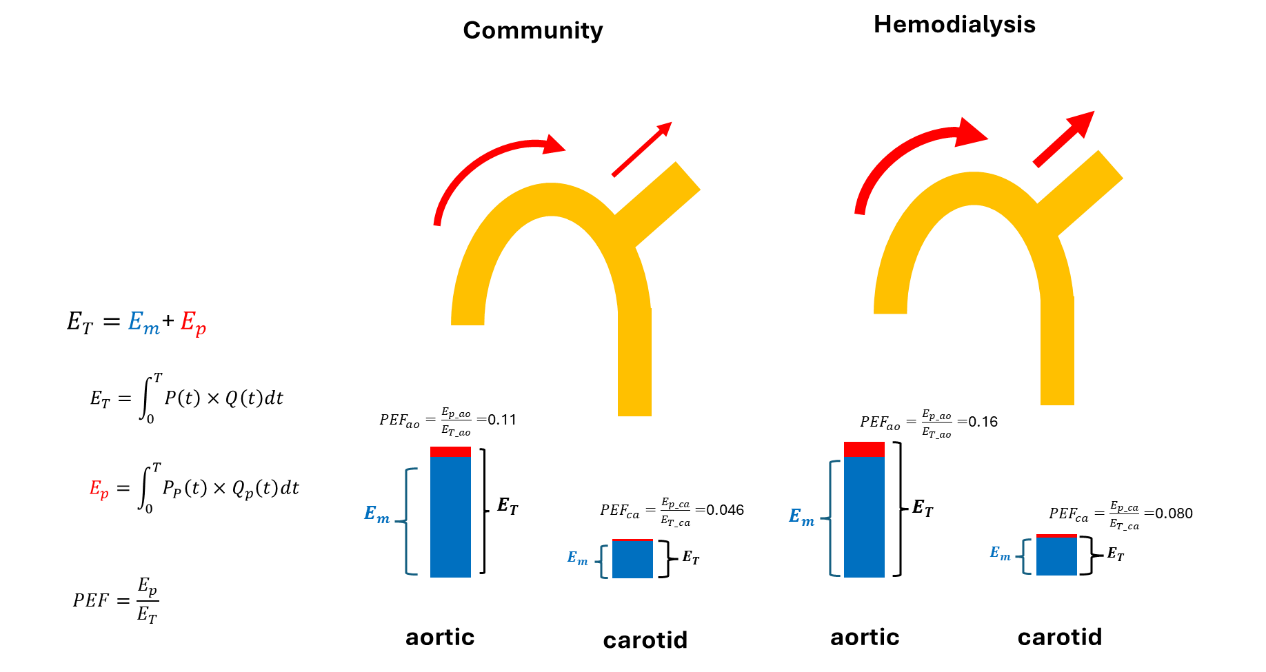


The total energy ($E_{T}$​) consists of mean energy ($E_{m}$) and pulsatile energy ($E_{p}$​),which are calculated by integration over one cardiac cycle. PEF is defined as the ratio $\frac{E_{p}}{E_{T}}$ representing the relative contribution of pulsatile energy. Though the mean energy in hemodialysis patients is relatively comparable to that in community cohort, the pulsatile energy is much larger in hemodialysis patients (0.16 vs. 0.11) and easier for the pulsatile energy to be transmitted into carotid artery (0.080 vs. 0.046)

**Supplementary Table 1** Baseline characteristics of the study participants

| **Variables**  **Mean (SD), n (%),or**  **median (IQR)** | **Hemodialysis**  **Cohort** | | **Community**  **Cohort** | | **P value** | |
| --- | --- | --- | --- | --- | --- | --- |
| **N** | 162 | | 1858 | |  | |
| Age, y | 64 (12) | | 61 (11) | | 0.001 | |
| Male, n (%) | 98 (61) | | 675 (36) | | <0.001 | |
| BMI, kg/m^2^ | 24.4(4.4) | | 24.0 (3.3) | | 0.081 | |
| Hemodialysis duration, y | 4.5(1.2,8.3) | | NA | |  | |
| **Medical history, n(%)** |  |  |  |  |  | |
| Hypertension | 132(81) | | 292 (16) | | <0.001 | |
| Diabetes mellitus | 90(56) | | 166 (9) | | <0.001 | |
| **Education level, n( %)** |  |  |  |  | <0.001 | |
| Elementary school/below | 25(15) | | 122 (7) | |  | |
| Junior school | 36(22) | | 171 (9) | |  | |
| High school | 66(41) | | 578 (31) | |  | |
| University or higher | 35(22) | | 991 (53) | |  | |
| **Biochemistry data** |  |  |  |  |  | |
| Cholesterol, mg/dl | 150 (38) | | 142 (35) | | <0.001 | |
| Albumin, g/dl | 3.9 (0.3) | | NA | |  | |
| Calcium x Phosphate | 48.2 (12.6) | | NA | |  | |
| Kt/V | 1.6 (0.3) | | NA | |  | |
| **Brachial BP** |  |  |  |  |  | |
| Systolic BP, mmHg | 134 (27) | | 125 (18) | | <0.001 | |
| Diastolic BP, mmHg | 75 (16) | | 75 (10) | | 0.787 | |
| Pulse pressure, mmHg | 58 (17) | | 50 (12) | | <0.001 | |
| Heart rate, /min | 71 (23) | | 70 (12) | | 0.175 | |
| **Blood vessel function** |  |  |  |  |  | |
| cfPWV, m/sec | 13.3 (5.8) | | 12.2 (3.6) | | 0.001 | |
| Zao, dyne*s/cm^5^ | 256 (170) | | 125 (62) | | <0.001 | |
| Zca, dyne*s/cm^5^ | 1493 (1769) | | 1162 (565) | | <0.001 | |
| **Cognitive function** |  |  |  |  |  | |
| Cognitive dysfunction, n(%) | 60(37.0) | | 429 (23.1) | | <0.001 | |
| MoCA score, median (IQR) | 26(23, 28) | | 27 (26, 29) | | <0.001 | |
| BP, blood pressure; cf-PWV, carotid-femoral pulse wave velocity; Kt/V, urea clearance; | | | | | |  |
| MoCA, Montreal Cognitive Assessment; Cognitive dysfunction: MoCA score<26 | | | | | |  |
| Values are expressed by n (%), mean (SD), or median (IQR) as appropriate | | | | | |  |

**Supplementary Table 2** Univariable associations of MoCA score with baseline clinical characteristics

| Variables | β | *P* value |  |
| --- | --- | --- | --- |
| Age, yr | -0.461 | 0.000 |  |
| Male gender | -0.040 | 0.624 |  |
| Body mass index | 0.163 | 0.045 |  |
| Hemodialysis duration, year | 0.001 | 0.992 |  |
| Education level | 0.186 | 0.022 |  |
| Hypertension | -0.040 | 0.628 |  |
| Diabetes mellitus | -0.092 | 0.262 |  |
| Albumin | 0.077 | 0.350 |  |
| Cholesterol | 0.008 | 0.927 |  |
| Hemoglobin | -0.117 | 0.157 |  |
| Calcium x phosphate | 0.091 | 0.278 |  |
| Kt/V | -0.096 | 0.260 |  |

Values are standardized β coefficients and p-values from univariable linear regression models with MoCA score as the dependent variable.

**Supplementary Table 3** Sensitivity analyses of the association between hemodynamic parameters and MoCA score after excluding patients with prior stroke and after additional adjustment for shunt side

| **Variables** | **Sensitivity analysis 1 ( exclude stroke)** | |  | **Sensitivity analysis 2**  **(add shunt side)** | |
| --- | --- | --- | --- | --- | --- |
|  | β | *P* value |  | β | *P* value |
| **cf-PWV** | -0.146 | 0.060 |  | -0.161 | 0.038 |
| **Brachial artery** |  |  |  |  |  |
| Systolic pressure, mmHg | -0.103 | 0.184 |  | -0.133 | 0.082 |
| Diastolic pressure, mmHg | -0.013 | 0.876 |  | -0.041 | 0.623 |
| Mean pressure, mmHg | -0.062 | 0.854 |  | -0.092 | 0.250 |
| Pulse pressure, mmHg | -0.147 | 0.053 |  | -0.173 | 0.021 |
| **Ascending aorta** |  |  |  |  |  |
| Mean flow, ml/s | 0.034 | 0.665 |  | 0.026 | 0.741 |
| Peak flow, ml/s | -0.082 | 0.302 |  | -0.077 | 0.333 |
| Flow pulsatility index | -0.018 | 0.811 |  | 0.013 | 0.867 |
| Total energy, MJ | 0.107 | 0.149 |  | 0.087 | 0.245 |
| Mean energy, MJ | 0.136 | 0.066 |  | 0.116 | 0.121 |
| Pulsatile energy, mJ | -0.042 | 0.589 |  | -0.053 | 0.486 |
| Pulsatile energy fraction | -0.181 | 0.029 |  | -0.176 | 0.032 |
| **Common carotid arteries** |  |  |  |  |  |
| Mean flow, ml/s | 0.150 | 0.062 |  | 0.116 | 0.153 |
| Peak flow, ml/s | -0.149 | 0.049 |  | -0.147 | 0.053 |
| Flow pulsatility index | -0.138 | 0.086 |  | -0.126 | 0.115 |
| Total energy, MJ | 0.172 | 0.021 |  | 0.139 | 0.066 |
| Mean energy, mJ | 0.190 | 0.011 |  | 0.157 | 0.038 |
| Pulsatile energy, mJ | -0.008 | 0.913 |  | -0.036 | 0.634 |
| Pulsatile energy fraction | -0.292 | <0.001 |  | -0.288 | <0.001 |

Values are standardized β coefficients and p-values from multivariable linear regression models with MoCA score as the dependent variable. All models were adjusted for age, sex, body mass index, and education level. Sensitivity analyses were performed (i) after excluding patients with prior stroke (n = 147) and (ii) with additional adjustment for shunt side (left vs right arm vascular access). Results were consistent with the primary analysis
